# Safety of anti-immunoglobulin E therapy with omalizumab in allergic patients at risk of geohelminth infection

**DOI:** 10.1111/j.1365-2222.2007.02650.x

**Published:** 2007-02-01

**Authors:** A A Cruz, F Lima, E Sarinho, G Ayre, C Martin, H Fox, P J Cooper

**Affiliations:** * ProAR, Faculdade de Medicina da Bahia-UFBA, Instituto de Investigação em Imunologia (iii) CNPq, Salvador, BA, Brazil; † Departamento de Pediatria, Faculdade de Medicina da Universidade Federal de Pernambuco Recife, Pernambuco, Brazil; ‡ Novartis Horsham Research Centre Horsham, UK; § Laboratorio de Investigaciones, Hospital Pedro Vicente Maldonado Pichincha Province, Ecuador

**Keywords:** allergic rhinitis, asthma, geohelminth infections, immunoglobulin E, monoclonal antibody, omalizumab

## Abstract

**Background:**

Although the role of immunoglobulin E (IgE) in immunity against helminth parasites is unclear, there is concern that therapeutic antibodies that neutralize IgE (anti-IgE) may be unsafe in subjects at risk of helminth infection.

**Objective:**

We conducted an exploratory study to investigate the safety of omalizumab (anti-IgE) in subjects with allergic asthma and/or perennial allergic rhinitis at high risk of intestinal helminth infection. The primary safety outcome was risk of infections with intestinal helminths during anti-IgE therapy.

**Methods:**

A randomized, double-blind, placebo-controlled trial was conducted in 137 subjects (12–30 years) at high risk of geohelminth infection. All subjects received pre-study anthelmintic treatment, followed by 52 weeks' treatment with omalizumab or placebo.

**Results:**

Of the omalizumab subjects 50% (34/68) experienced at least one intestinal geohelminth infection compared with 41% (28/69) of placebo subjects [odds ratio (OR) 1.47, 95% confidence interval (CI) 0.74–2.95, one-sided *P* = 0.14; OR (adjusted for study visit, baseline infection status, gender and age) 2.2 (0.94–5.15); one-sided *P* = 0.035], providing some evidence for a potential increased incidence of geohelminth infection in subjects receiving omalizumab. Omalizumab therapy was well tolerated, and did not appear to be associated with increased morbidity attributable to intestinal helminths as assessed by clinical and laboratory adverse events, maximal helminth infection intensities and additional anthelmintic requirements. Time to first infection (OR 1.30, 95% CI 0.79–2.15, one-sided *P* = 0.15) was similar between treatment groups. Infection severity and response to anthelmintics appeared to be unaffected by omalizumab therapy.

**Conclusions:**

In this exploratory study of allergic subjects at high risk of helminth infections, omalizumab therapy appeared to be safe and well tolerated, but may be associated with a modest increase in the incidence of geohelminth infection.

## Introduction

Intestinal helminth infections caused by *Ascaris lumbricoides*, hookworm, *Trichuris trichiura* and *Strongyloides stercoralis* are the most prevalent helminth parasitic infections in humans, infecting approximately 2 billion people worldwide [1]. The vast majority of helminth infections do not lead to associated clinical morbidity even in those areas where such infections are a public health concern [2]. Helminth antigens are potent inducers of immunoglobulin E (IgE) production and are capable of stimulating an IgE response in almost all infected individuals [3]. Although levels of IgE increase substantially with persistent exposure to helminth parasites, the role of IgE in mediating a protective anti-helminth immune response remains controversial [4]. Animal studies indicate that immune responses to helminths involve multiple effector pathways directed against different stages of the helminth life cycle [5]. Current evidence in humans indicates that protective immunity against intestinal helminths is mediated by type-2 cytokine immune responses [6]. There is little consistent evidence to suggest that antibodies of any particular isotype or subclass represent a primary effector mechanism against these parasites [4].

Omalizumab (Xolair®, Novartis Pharmaceuticals Corporation) is a novel humanized anti-IgE monoclonal antibody licensed for the treatment of allergic asthma (AA). In clinical studies, omalizumab has been shown to provide effective control of AA and perennial allergic rhinitis (PAR) [7–14]. Omalizumab binds IgE and inhibits binding to high-affinity (FcåRI) IgE receptors on mast cells and basophils, thereby reducing mediator release at the start of allergic responses [15].

There is concern that neutralizing IgE may adversely affect immunity to helminth parasites. To further characterize the safety profile of omalizumab, we need to determine whether treatment is safe in patients exposed to intestinal helminth infection and whether susceptibility to infection is increased, particularly in patients who travel to or live in areas in which these infections are endemic.

We conducted an exploratory study using a randomized, placebo-controlled design to investigate three specific issues relating to the safety of omalizumab treatment in allergic individuals at risk of intestinal helminth infections: (1) the incidence of intestinal helminth infection; (2) clinical and laboratory adverse events (AEs) that could be associated with disease caused by intestinal helminths and other safety parameters associated with infection; and (3) the effect of treatment on time to infection.

## Methods

### Subjects

Males and females aged 12–30 years with a diagnosis of AA for ≥1 year and/or a clinical history of PAR for >2 years, a body weight of 20–150 kg, platelet levels of at least 130 × 10^9^/L and either a current geohelminth infection (any of *A. lumbricoides*, *T. trichiura*, hookworm, *S. stercoralis* or *Enterobius vermicularis*) or a high risk of infection were enrolled. ‘High risk of infection’ was defined as having had at least one of the following: documented history of geohelminth infection or living with a household member with a current or documented history of geohelminth infection within the previous year, or a positive RAST for Ascaris-specific IgE at screening and a self-reported geohelminth infection within the previous year. Self-reporting of infection was always validated by medical records or RAST. The exclusion criteria were as follows: total IgE ≥1300 IU/mL; weight ≤20 kg or >150 kg; weight-to-IgE ratios that fell outside values that permitted dosing as set out in the dosing table; a history of severe anaphylactoid or anaphylactic reaction(s); use of oral corticosteroids or high dose of inhaled corticosteroids (>500 μg fluticasone or >800 μg budesonide per day) within 4 weeks of the study or likelihood of needing regular-inhaled corticosteroids at such doses during the study; and use of methotrexate, gold salts, cyclosporine or troleandomycin within 3 months of the study.

Ethical approval was obtained from the institutional review boards of each study centre and by the Brazilian National Commission for Ethics in Research (CONEP). All subjects (or their parents or guardians) gave written informed consent. The study was conducted in accordance with the principles of the Declaration of Helsinki and European Community and US Food and Drug Administration regulations. A Data Safety Monitoring Board analysed data during the course of the study to advise the clinical study team of any potential modifications to the study conduct necessary to minimize potential risks to subjects.

### Study design

This was a randomized, double-blind, parallel-group, placebo-controlled study conducted at seven urban centres in Brazil (Salvador, Curitiba, Rio de Janeiro, Goiania, Recife and two centres in Fortaleza). The study comprised a 4-week screening period, when subjects received anthelmintic treatment to clear intestinal and other helminth infections as well as routine care to treat asthma and PAR. This treatment could include the use of long-acting β_2_-agonists, inhaled or nasal steroids, leucotriene inhibitors/receptor antagonists or antihistamines. Subjects were not included if they required >500 μg fluticasone or >800 μg budesonide/beclometasone per day on a regular basis to avoid potential confounding effects of the potential albeit unlikely broad immunosuppressive actions of these medicines that could lead to an increased susceptibility to geohelminth infection. Subjects receiving allergen-specific subcutaneous immunotherapy were also eligible, but such a treatment could not be initiated during the study. The screening period was followed by a 52-week treatment period, during which subjects received subcutaneous omalizumab or placebo, followed by albendazole treatment at the end of the 52 weeks and a 12-week follow-up period.

### Randomization

Subjects were assigned to treatment groups using a randomization number provided by Novartis Drug Supply Management using a validated computerized system (Almedica Drug Labeling System, Version 5.3 a, Almedica Technology Group Inc., Allendale, NJ, USA). Blocks of four randomization numbers were allocated to each treatment centre. Investigators and personnel in the Quality Assurance Biostatistics Group in the Novartis Biostatistics and Statistical Reporting Group held randomization details and were authorized to unmask individual subject assignments only in case of emergency. Treatment allocation was unblinded after the study had been completed and all data had been computer entered and locked.

### Intervention

Omalizumab dosage and dosing frequency was calculated based on body weight and total serum IgE levels measured at screening. Subjects received either omalizumab 150 or 300 mg every 4 weeks or 225, 300 or 375 mg every 2 weeks. The amount of omalizumab to administer, and whether to dose every 2 or every 4 weeks, is based on patient body weight and IgE level set out in a dosing table (Appendix A1). Open-label omalizumab or placebo supplies were shipped to each site and reconstituted by a specified individual who was instructed not to reveal the identity of the drug to the subjects, investigator or Novartis personnel involved in conducting, monitoring or analysing the data in this trial.

### Anthelmintic treatment

All subjects were treated with broad-spectrum anthelmintic drugs before the start of the study: a single dose of 400 mg of albendazole, followed by a repeat dose of albendazole plus 150 μg/kg of ivermectin 14 days later (i.e. 7 days before the dose of study drug). Ivermectin would be expected to have >95% cure rates for *S. stercoralis* [15] and two doses of albendazole would be expected to have >95% cure rate for other intestinal helminth infections, except *T. trichiura* [15]. The combination of albendazole/ivermectin is more efficacious than either drug alone for infections with *T. trichiura* [16]. Subjects with schistosomiasis were treated with a standard dose of praziquantel or oxamniquine. During the treatment period, anti-parasite therapy was initiated if the measured eggs per gram (epg) of faeces for a particular parasite was above the following pre-defined safety thresholds: *A. lumbricoides*, 1000; hookworm, 200; *T. trichiura*, 1000; and *Schistosomiasis mansoni*, 500. *Strongyloides* infection was treated with one dose of ivermectin and the subject was withdrawn from the study at the next visit if the infection persisted. At the end of week 52 of treatment, all subjects received anthelmintic treatment.

### Subject assessments and laboratory analyses

The following assessments were undertaken every 4 weeks during the treatment and follow-up periods: faeces sampling, spirometry, vital signs, physical examination, pregnancy testing and recording of AEs. Routine laboratory assessments for haematology, clinical chemistry and urinalysis were performed every 12 weeks.

**Fig. 1:**
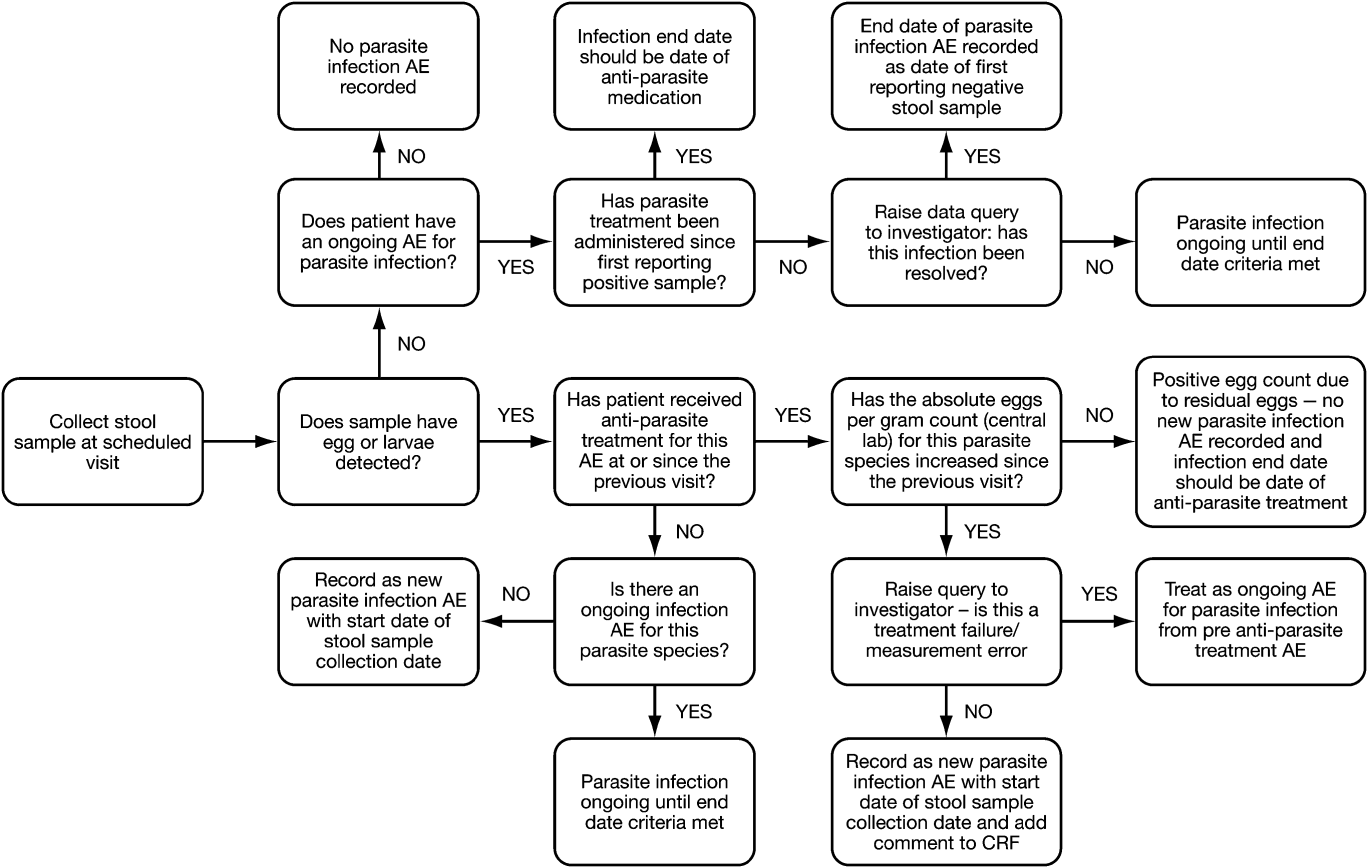
Flow chart for determining intestinal helminth infection status. Intestinal helminth infections were defined as new infections if helminth eggs were detected in a faeces sample following a previously negative sample, or there was an increase in infection intensity [eggs per gram (epg)] for the same species at least 4 weeks after appropriate anthelmintic treatment or an increase in infection intensity (epg) for the same parasite species compared with the previous sample. The start date of a new infection was defined as the date on which the positive sample was taken. The end of a helminth infection was defined as the date on which either (1) anthelmintic treatment was given to treat a specific helminth infection or (2) no eggs/larvae were detected and the investigator considered the infection resolved or (3) when anthelmintic treatment was administered at the end of the 52-week treatment period.

Faeces samples were examined at a central laboratory (Fleury, Brazil) using both a direct wet mount technique [17] and the modified Kato–Katz method [17] for the detection and quantification of intestinal helminth infections and the Baermann method for detection of *Strongyloides* larvae [18]. A parasite infection identification flow chart was used in the study and is shown in Fig. 1. Infection intensity was quantified by egg counts and reported as epg of faeces. Faeces samples were also analysed using appropriate methods at local laboratories to ensure a rapid turnaround of samples. Local sampling facilities were used for determination of positive helminth infections, but not for quantification. For standardization purposes, all egg count values reported are from the central laboratory. In addition, serology was performed before randomization in a subset of subjects at a single centre (Salvador) to detect prior exposure to *S. stercoralis*, using an ELISA to detect specific IgG [19], and reactions were classified as positive, equivocal or negative.

### Outcome variables

The primary safety outcome was the rate of intestinal geohelminth parasite infection. The time to first detection of infection with intestinal geohelminth parasites was also evaluated. The classification of intestinal helminth infection status during the study is described in Fig. 1.

Other safety outcomes were clinical AEs, relevant laboratory measurements (peripheral eosinophil counts, liver function and haemoglobin), maximal helminth infection intensities during the study and requirement for additional anthelmintic treatments. Clinical AEs were graded as mild, moderate or severe. Serious AEs were also recorded and were defined as events that were fatal or life-threatening, required hospitalization, were significantly or permanently disabling or incapacitating, constituted a congenital anomaly or a birth defect or were considered medically significant.

### Statistical analysis

The study was exploratory and the planned sample size of 70 subjects per treatment arm was based primarily on practical considerations. From this practical perspective, identifying allergic subjects at high risk of geohelminth infection who had IgE levels within the inclusion criteria limits was always likely to prove challenging. The population targeted for this study was selected to allow a meaningful number of infection events to be evaluated in an experimental context. Seventy patients per arm were considered to provide important safety data in this group of subjects, and would allow large effects on geohelminth infection to be detected.

The geohelminth infection rates were analysed using logistic regression, with treatment, dose schedule and study centre as covariates. A secondary analysis was performed to adjust for additional variables including those for which imbalances were present at baseline and adjusted for study visit, baseline infection status, gender and age. Time to infection with intestinal geohelminths was estimated using Kaplan–Meier analyses to evaluate infection-free status within each treatment group and the relative risk (RR) calculated using a Cox's regression model with dosing schedule and pooled centre as covariates.

One-sided *P*-values in favour of placebo were presented for omalizumab relative to placebo, with *P* < 0.025 suggesting excessive risk and *P* > 0.10 suggesting no excessive risk. The study was not powered for formal hypothesis testing of geohelminth reinfection risk, and one-sided *P*-values were presented as a safety signal for an increase in the incidence of infections with omalizumab relative to placebo.

## Results

### Baseline characteristics and subject follow-up

**Fig. 2:**
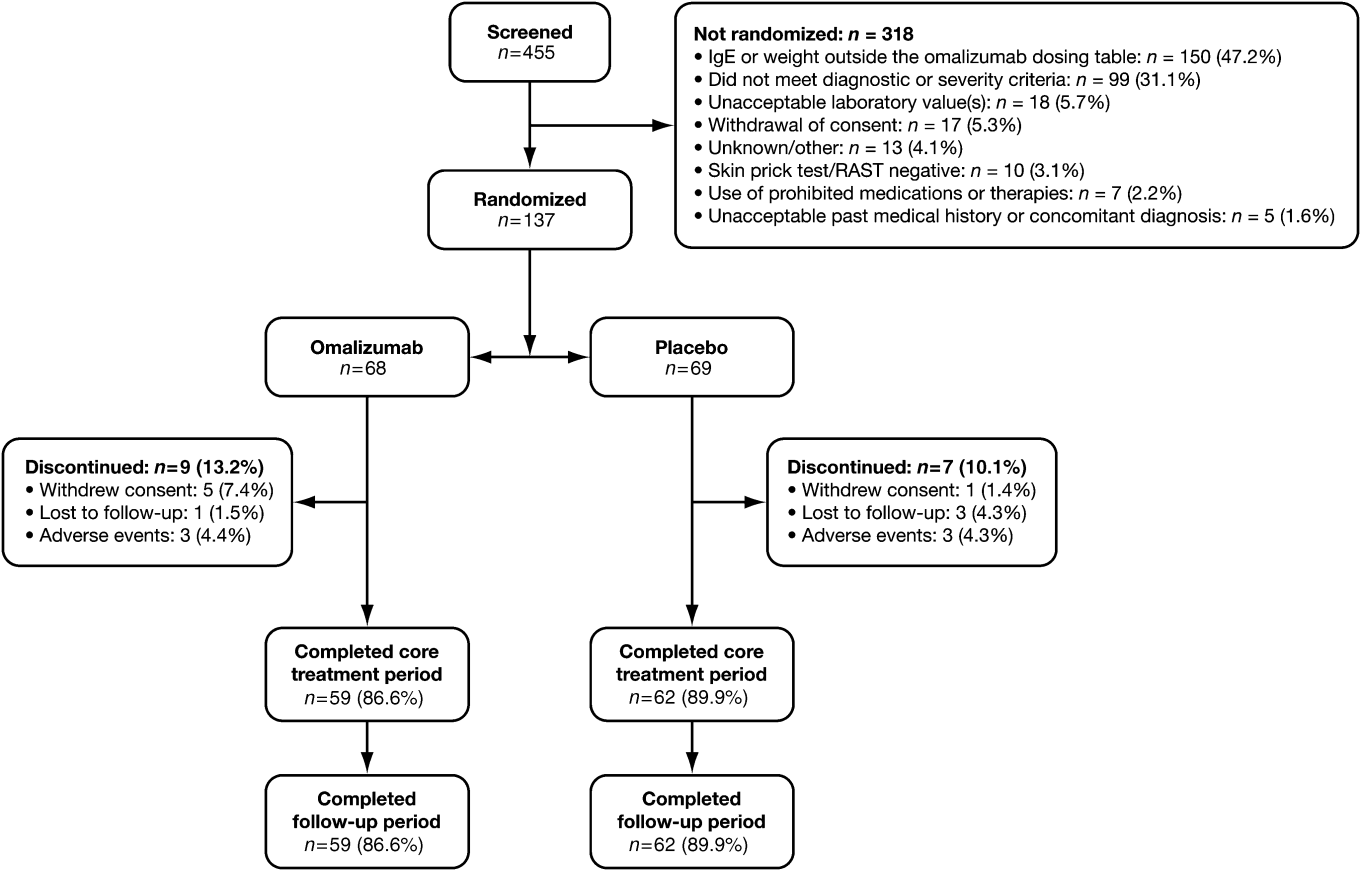
Flow of subjects through the study.

This study was conducted between November 2001 and March 2004. A total of 455 subjects were screened and 137 were randomized (omalizumab, *n* =68; placebo, *n* =69; Fig. 2). Treatment and follow-up periods were completed by 88% (121/137) of subjects (omalizumab, 59/68: placebo, 62/69). There were non-statistically significant but relevant baseline imbalances between the two groups for the frequency of active infections in the placebo group (40.6% vs. 27.9%) and a greater proportion of males in the omalizumab group than in the placebo group (48.5% vs. 36.2%; Table 1). Treatment groups were balanced for the distribution of subjects between dosing regimens of the study drug (data not shown). All subjects were free of intestinal geohelminth infection at the start of the 52-week treatment period.

**Table 1:** Summary of baseline demographics and clinical characteristics

	Omalizumab (*n* =68)	Placebo (*n* =69)
Males, *n* (%)	33 (48.5)	25 (36.2)
Median age (years; range)	15 (12–29)	16 (12–30)
Median total serum IgE (IU/mL; range)	403 (39–1100)	397 (50–1231)
Median FEV_1_ (% predicted; range)	68 (33–105)	78 (40–103)
Allergy history		
AA, *n* (%)	48 (70.6)	52 (75.4)
Median duration of AA, years (range)	11 (0–26)	12 (1–24)
PAR patients, *n* (%)	64 (94.1)	65 (94.2)
Median duration of PAR, years (range)	6 (0–26)	8 (1–21)
Infection status at screening		
Active infection*, *n* (%)	19 (27.9)	28 (40.6)
History of any intestinal helminth infection in previous year, *n* (%)	43 (63.2)	53 (76.8)
Positive RAST test for *Ascaris*-specific IgE, *n* (%)	7 (10.3)	7 (10.1)
*Strongyloides stercoralis* infection† (*n*)	2	9 (13.0)
Household member with documented active/history of any intestinal helminth infection in previous year, *n* (%)	18 (26.5)	

* Based on positive egg count in faeces sample collected at screening.

† Serology for *S. stercoralis* was performed in a subset of 51 subjects. Equivocal results were obtained in another five subjects (3/26 with omalizumab and 2/25 with placebo).

IgE, immunoglobulin E; FEV_1_, forced expiratory volume in 1 second; AA, allergic asthma; PAR, perennial allergic rhinitis.

### Intestinal geohelminth infections

Over the treatment period, 50% (34/68) of omalizumab subjects experienced at least one intestinal geohelminth infection compared with 41% (28/69) of placebo subjects. There appeared to be a greater risk of intestinal helminth infection in the omalizumab group during the 52-week treatment period [odds ratio (OR) 1.47, 95% confidence interval (CI) 0.74–2.95, one-sided *P* =0.14], although the 95% CI is consistent with no effect (i.e. OR of 1). When adjusted for study visit, baseline infection status, gender and age, the OR was 2.2 (95% CI 0.94–5.15; one-sided *P* =0.035).

**Fig. 3:**
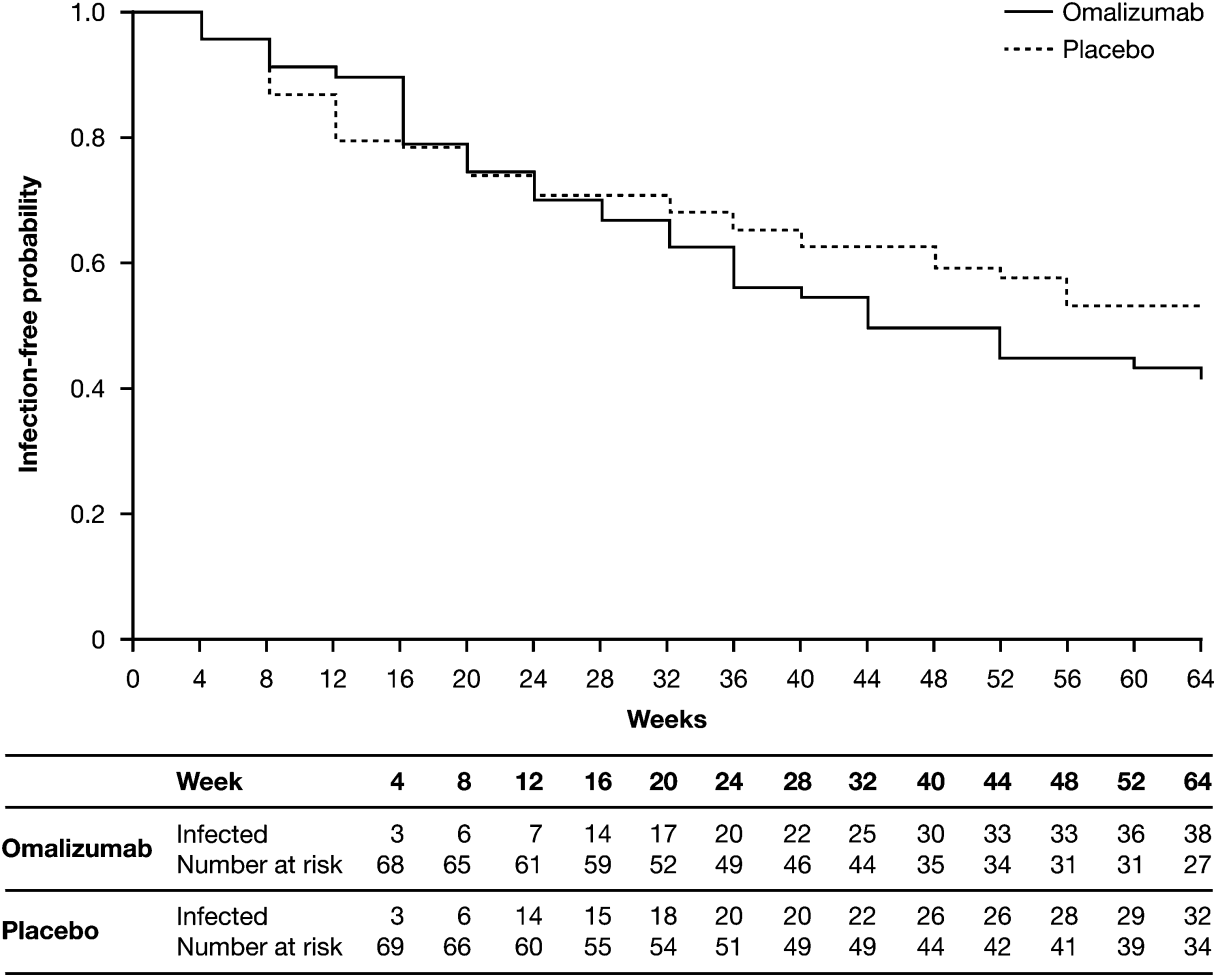
Kaplan–Meier plot of time to first detection of infection with intestinal helminth parasite during core and follow-up phases. These data include data for discontinued patients, so the number of patients infected in this analysis (36 vs. 29) differs slightly from the number of reported infections during the 52-week treatment period (34 vs. 28).

Time to first intestinal helminth infection after anthelmintic treatment provided during the initial screening phase of the study was similar in omalizumab and placebo groups [RR for treatment effect, 1.3 (95% CI 0.79–2.15; one-sided *P* = 0.150)]. The number of geohelminth infections by time and the Kaplan–Meier plot for time to first detection of infection are shown in Fig. 3, and indicate a possible trend for increased infection risk in the omalizumab group after 28 weeks of treatment.

The total number of subjects experiencing each type of geohelminth infection is presented in Table 2. Multiple new infections with *A. lumbricoides* were observed in two of 68 (2.9%) individuals in the omalizumab group but no multiple infections were observed in the placebo group.

**Table 2:** Number of subjects with intestinal geohelminth infection by helminth type during the 52-week treatment period

	Omalizumab (*n* =68)		Placebo (*n* =69)	
		
	Total number of subjects with infection, *n* (%)	Total number of infections	Total number of subjects with infection, *n* (%)	Total number of infections
Geohelminth infection*	34 (50.0)	60	28 (40.6)	42
*Ascaris lumbricoides*	21 (30.9)	28	16 (23.2)	16
Hookworm†	5 (7.4)	5	8 (11.6)	8
*Trichuris trichiura*	15 (22.1)	15	9 (13.0)	10
*Enterobius vermicularis*	11 (16.2)	12	6 (8.7)	6
*Strongyloides*	0	0	2 (2.9)	2
Other helminth infections	4 (5.9)	4	3 (4.3)	3
*Hymenolepis nana*	0	0	2 (2.9)	2
*Schistosoma mansoni*	4 (5.9)	4	1 (1.4)	1
Enteric protozoa infections	34 (50.0)	47	31 (44.9)	43

* *P* = 0.06 for overall comparison of geohelminth infection rate (repeated measures logistic regression with treatment, dosing schedule, centre and study visit as factors).

† *Ancylostoma* and *Necator* spp. Protozoa identified were *Blastocystis hominis, Chilomastix mesnili, Endolimax nana, Entamoeba coli, Entamoeba histolytica, Giardia intestinalis and Iodamoeba butsc*.

The clinical severity of parasite infections was similar between treatment groups. None of the parasite infections experienced by subjects in either treatment group was considered to be clinically severe by the investigators at the study centres.

### Clinical adverse events

AEs could be attributed to the study drug or intestinal helminth infections. The distribution of AEs was similar between treatment groups, with the exception of a higher incidence of skin and subcutaneous tissue disorders in the omalizumab group compared with the placebo group (Table 3). The incidence of urticaria (which could potentially be attributed to intestinal helminth infections) was very low in both treatment groups (one subject in each group). *Severe* AEs were reported in five omalizumab-treated subjects and four placebo recipients (Table 3). Three *serious* AEs, i.e. events that were fatal or life-threatening, required hospitalization, were significantly or permanently disabling or incapacitating, constituted a congenital anomaly or a birth defect or were considered medically significant, were reported (one omalizumab and two placebo; Table 3). No severe AE or serious AE was considered to be treatment related. Three subjects left the study due to AEs in each treatment group (placebo: pregnancy; tuberculosis; omalizumab: pregnancy). None of the events were considered treatment related.

**Table 3:** Number (%) of subjects with adverse events during the 52-week treatment period

Adverse events	Omalizumab (*n* = 68) *n* (%)	Placebo (*n* = 69) *n* (%)
Any adverse events	68 (100)	69 (100)
Suspected by investigator to be drug related	16 (23.5)	22 (31.9)
Severity		
Mild	28 (41.2)	25 (36.2)
Moderate	35 (51.5)	40 (58.0)
Severe*	5 (7.4)	4 (5.8)
Serious adverse events†	1 (1.5)	2 (2.9)
System organ class affected (≥5% in either treatment group)		
Infections and infestations	66 (97.1)	68 (98.6)
Respiratory, thoracic and mediastinal disorders	48 (70.6)	60 (87.0)
Nervous system disorders	38 (55.9)	50 (72.5)
Gastrointestinal disorders	35 (51.5)	39 (56.5)
Skin and subcutaneous tissue disorders	30 (44.1)	15 (21.7)
General disorders and administration site conditions	21 (30.9)	22 (31.9)
Reproductive system and breast disorders	17 (25.0)	17 (24.6)
Musculoskeletal and connective tissue disorders	14 (20.6)	20 (29.0)
Injury, poisoning and procedural complications	14 (20.6)	11 (15.9)
Eye disorders	10 (14.7)	17 (24.6)
Ear and labyrinth disorders	5 (7.4)	3 (4.3)
Blood and lymphatic system disorders	3 (4.4)	4 (5.8)
Metabolism and nutrition disorders	3 (4.4)	4 (5.8)
Vascular disorders	1 (1.5)	4 (5.8)

* Three omalizumab-treated subjects had a single severe adverse event (AE; elective abortion, headache, rhinitis) and 2 subjects had 2 severe AEs (chest pain/headache and anxiety/headache). One placebo recipient had 3 severe AEs (pregnancy, spontaneous abortion, giardiasis) and 3 subjects had a single severe AE (abdominal pain, hypotension, asthma).

† Elective abortion (omalizumab-treated patient), spontaneous abortion (placebo) and severe asthma exacerbation (placebo).

### Laboratory evaluations

Laboratory analyses revealed few haematological or biochemical changes, with no clinically or statistically relevant differences between treatment groups. Of particular interest were AEs that could be associated with geohelminth infections: low haemoglobin (intestinal blood loss caused by adult worms); eosinophilia (parasite infection); and elevation of alkaline phosphatase (ALP) and the liver transaminases alanine aminotransferase (ALT) and aspartate aminotransferase (AST; migration of helminth larvae in the liver/liver function). The median baseline values and mean change from baseline at 52 weeks for these parameters were similar in the omalizumab and placebo group (Table 4). One omalizumab-treated subject [1/68 (1.5%)] and one placebo recipient [1/69 (1.4%)] had abnormally low haemoglobin levels (male: <11.5 g/dL; female <9.5 g/dL) during the study. A trend towards more frequent abnormally elevated levels of ALP and liver transaminases was observed in omalizumab-treated subjects vs. placebo recipients; ALT: omalizumab 11.8% (8/68) vs. placebo 7.2% (5/69); AST: omalizumab 10.3% (7/68) vs. placebo 7.2% (5/69) and ALP: omalizumab 20.6% (14/68) vs. placebo, 8.7% (6/69). Ten of the 14 subjects with elevated ALP in the omalizumab group had an intestinal helminth infection during the study compared with three of the six placebo recipients. However, two of the 14 omalizumab and none of the six placebo-treated subjects had elevated ALP levels at baseline. ALT and AST levels were similar irrespective of geohelminth infection in the two groups.

**Table 4:** Summary of changes in laboratory-evaluated biochemical parameters during the 52-week treatment period

	Omalizumab (*n* =68)		Placebo (*n* =69)	
		
	Mean change from baseline at 52 weeks	Median at baseline (range)	Mean change from baseline at 52 weeks	Median at baseline (range)
Parameter (units)				
Haemoglobin (g/dL)	−0.1	13.5 (11.3–16.0)	−0.1	13.5 (9.8–17.8)
Eosinophils (%)	−1.6	5.6 (1.4–26.5)	−0.4	5.6 (0.9–21.9)
Alanine aminotransferase, ALT (U/L)	−0.3	15.0 (9–44)	−0.9	14.0 (7–78)
Alkaline phosphatase (serum), ALP (U/L)	−38.5	456.5 (92–1.238)	−40.0	305 (100–1.002)
Aspartate aminotransferase, AST (U/L)	−0.7	22.5 (13–44)	−0.3	22.0 (13–47)

### Clinical intervention for intestinal helminth infections

During the study, anthelmintic treatments were provided if infection intensities increased above pre-defined thresholds. Similar numbers of subjects in the omalizumab- and placebo-treated groups experienced geohelminth infections exceeding the thresholds during core treatment plus follow-up periods (15 infections in nine subjects in the omalizumab group compared with 12 infections in 11 subjects in the placebo group). One subject in the omalizumab group experienced five infections that exceeded the epg of faeces threshold caused by roundworm (three infections), hookworm (one infection) and whipworm (one infection). Two additional subjects in the omalizumab-treated group received anthelmintic treatments in error with lower egg counts than required by the study protocol.

All appropriate anthelmintic treatments administered for infections with *A. lumbricoides*, hookworm and *S. stercoralis* infections during the study resulted in a cure in both intervention groups. One subject in the omalizumab group was treated with a single 400 mg dose of albendazole for whipworm infection, which reduced the egg count below threshold but did not eliminate infection. However, albendazole has a relatively low cure rate for the treatment of whipworm [20].

### Peak infection intensities

There was a wide variation in the intensity of intestinal helminth infection. The median peak infection intensities were similar between omalizumab and placebo-treated infected subjects: *A. lumbricoides* [omalizumab, median 432 epg (range 24–29, 160) vs. placebo 1248 epg (range 24–11 328)]; *T. trichiura* [omalizumab, median 108 epg (range 48–1272) vs. placebo 60 (range 24–624)].

## Discussion

We performed a randomized-controlled trial to evaluate the safety of omalizumab in allergic subjects considered to be at a high risk of infection with intestinal helminths. Our findings provide evidence that omalizumab can be safely administered to such patients but do not exclude a possible increased risk of helminth infection in patients at a high risk of infection. Because of the wide CIs for this, our findings are consistent with a possible slightly increased risk of infection, although this failed to reach statistical significance.

We do not believe that a possible increased risk of infection with common intestinal helminth parasites is likely to be of clinical significance because no excess clinical or relevant laboratory AEs were reported in the omalizumab-treated group compared with placebo, with the exception of: (1) a higher incidence of skin and subcutaneous tissue disorders in the omalizumab group that was related largely to injection site reactions and (2) a trend towards more frequent abnormally elevated liver enzymes that did not appear to be associated with intestinal helminth infection. Peak infection intensities and requirement for additional anthelmintic treatments during the course of the study were comparable between treatment groups.

The main limitation of this exploratory study was the relatively small sample size and therefore the low power to detect a small increase, for the primary safety outcome, in the risk of geohelminth infection. Based on the sample size (approximately 70 subjects per treatment arm) and the observed variance in the study, retrospective calculations indicated that this exploratory study had an 80% power to detect a 28% increase in the incidence of at least one intestinal geohelminth infection in the omalizumab group, over the 41% encountered in the placebo group (0.05 significance level, two-sided). Assuming a similar treatment difference, it is possible to speculate that a statistically significant effect on risk of geohelminth infection may have been observed in a larger study. However, it is important to note that this is a high-risk population and that there was only one (0.03%) reported helminthic infection (pinworm) from 1934 patient-years of omalizumab treatment in 3678 treated patients in the omalizumab clinical trials programme. In addition, there have been no spontaneous post-marketing reports of helminth infection in the United States since launch in more than 39 000 patients. The population targeted for this study was selected to allow a meaningful number of infection events to be evaluated in an experimental context. This requirement led to severe recruitment problems as (1) heavily infected and exposed individuals (i.e. at highest risk of infection) had total IgE levels that were considerably in excess of the upper IgE limit for inclusion (≤1300 IU/mL); and (2) allergic individuals (with a disease profile likely to respond to omalizumab therapy) have a low risk of infection with geohelminth parasites. Recent studies have shown strong inverse relationships between intestinal helminth infections and atopy [18, 19] or asthma [19, 21], which may indicate that allergic individuals are more resistant to helminth infections. It would appear unlikely, therefore, that a larger randomized-controlled trial could be performed in the future, and the data from the present study are therefore important for the information they provide on safety.

We also assessed safety using several parasitologic parameters designed to detect the rapid acquisition of heavy helminth burdens (time to treatment with anthelmintics), a tendency towards higher helminth burdens (maximal infection intensities and requirement for additional anthelmintic treatments) and response to anthelmintic treatment (immune-dependent response). These parameters were similar in both treatment groups. Overall, the safety data suggest that omalizumab treatment is not associated with clinically relevant AEs that might be caused by helminth infection, and they provide evidence that omalizumab can be safely administered to allergic patients who travel to or work temporarily in areas that are endemic for intestinal helminths. The data also show that anthelmintic drugs can be administered to patients who are receiving omalizumab and who are infected with helminth parasites without a loss of therapeutic efficacy. This may not be surprising because the effects of anthelmintic drugs on adult worms in the intestine are not thought to depend on host immunity.

The study was conducted in allergic subjects considered to be at risk of intestinal helminth infection. The study population was, in many ways, somewhat atypical of individuals who live in highly endemic areas for geohelminth infection because of significant allergic disease and relatively low IgE levels (≤1300 IU/mL). Populations living in areas that are highly endemic for geohelminth parasites tend to have very high levels of polyclonal IgE [21], a low risk of allergic disease, with severe allergic disease being particularly rare [22]. The study population in the present study represents a population of individuals with allergic disease at risk of geohelminth infection through living in poor urban areas of Brazil.

The study provided a unique opportunity to examine the protective role of IgE against intestinal helminth parasites. There is a general belief that IgE is an important mediator of host immunity against helminth parasites [23–25], although much of the evidence is conflicting and derives from experimental animal models [26, 27]. The finding of a possible increased risk of intestinal helminth infection in individuals receiving omalizumab treatment is consistent with a protective role for this Ig. However, the estimated risks were relatively modest, lending support to observations from animal models that suggest a non-critical role for IgE in protective immunity [4]. There were too few infections with *S. stercoralis* (omalizumab 0 vs. placebo 2 infections) to allow an evaluation of safety in this study. *S. stercoralis* is unusual in that it is capable of replicating within the human host, and occasionally may cause a severe and potentially fatal condition, *Strongyloides* hyperinfection, which is associated with specific acquired immune deficiency disorders. Some concern for a potential increased risk of *Strongyloides* hyperinfection in patients taking omalizumab is justified, although the risk is likely to be no greater than with more broadly immunosuppressive treatments such as oral corticosteroids that have been associated with hyperinfection [28]. As when considering treatment with oral corticosteroids, specific groups of allergic individuals being considered for treatment with omalizumab and who are at a particularly high risk of *Strongyloides* infection should have a parasitologic assessment before the start of treatment to exclude active strongyloidiasis. Such individuals might include institutionalized psychiatric patients, the indigent (i.e. impoverished individuals lacking necessities such as food and shelter) and refugees or migrants from endemic areas. Individuals at a high risk of the hyperinfection syndrome and who have active *Strongyloides* infections should probably be excluded from omalizumab treatment, as would occur in individuals with haematologic malignancies and human T lymphotrophic virus type-1 (HTLV-1) co-infection [29].

In conclusion, this randomized-controlled trial provides evidence that omalizumab can be safely used to treat AA and PAR in patients considered to be at risk of common intestinal helminth infections. Because we were unable to obtain sufficient data on strongyloidiasis, we cannot exclude a safety risk for this infection, although the theoretical risk could be minimized by adequate screening of at-risk subjects, before the start of therapy. There was some evidence for a slightly increased incidence of intestinal helminth parasite infection among subjects receiving omalizumab, but this possible excess infection risk did not appear to be clinically significant.

## Appendix A1

Dosing tables for omalizumab administered by subcutaneous injection every 4 weeks (A) or every 2 weeks (B). Omalizumab doses are given in milligrams per dose. These dosing tables differ from the dosing tables provided in the package inserts of the marketed product.

**Appendix A1:** Dosing tables for omalizumab administered by subcutaneous injection every 4 weeks (A) or every 2 weeks (B)

	Bodyweight (kg)								
	
Baseline IgE (IU/mL)	>20–30	>30–40	>40–50	>50–60	>60–70	>70–80	>80–90	>90–125	>125–150
A									
≥30–100	150	150	150	150	150	150	150	300	300
>100–200	150	150	300	300	300	300	300		
>200–300	150	300	300	300					
>300–400	300	300							
>400–500	300						Administration every 2 weeks see Part B		
>500–600	300								
B									
≥30–100		Administration every 4 weeks see Part A							
>100–200								225	300
>200–300					225	225	225	300	375
>300–400			225	225	225	300	300		
>400–500		225	225	300	300	375	375		
>500–600		225	300	300	375				
>600–700	225	225	300	375					
>700–800	225	300	375						
>800–900	225	300	375						
>900–1000	300	375				DO NOT DOSE			
>1000–1100	300	375							
>1100–1200	300								
>1200–1300	375								

Omalizumab doses are given in mg/dose.

These dosing tables differ from the dosing tables provided in the package inserts of the marketed product.
